# Applying economic principles to health care.

**DOI:** 10.3201/eid0702.010227

**Published:** 2001

**Authors:** R D Scott, S L Solomon, J E McGowan

**Affiliations:** Centers for Disease Control and Prevention, Atlanta, Georgia 30333, USA. Dscott1@cdc.gov

## Abstract

Applying economic thinking to an understanding of resource use in patient care is challenging given the complexities of delivering health care in a hospital. Health-care markets lack the characteristics needed to determine a "market" price that reflects the economic value of resources used. However, resource allocation in a hospital can be analyzed by using production theory to determine efficient resource use. The information provided by hospital epidemiologists is critical to understanding health-care production processes used by a hospital and developing economic incentives to promote antibiotic effectiveness and infection control.


### Applying Economic Principles to Health Care

**R. Douglas Scott II,* Steven L. Solomon,* and John E. McGowan, Jr.†**
*Centers for Disease Control and Prevention, Atlanta, Georgia, USA;
and †Emory University School of Medicine, Atlanta, Georgia, USA

Applying economic thinking to an understanding of resource use in patient care is challenging given the complexities of delivering health care in a hospital. Health-care markets lack the characteristics needed to determine a "market" price that reflects the economic value of resources used. However, resource allocation in a hospital can be analyzed by using production theory to determine efficient resource use. The information provided by hospital epidemiologists is critical to understanding health-care production processes used by a hospital and developing economic incentives to promote antibiotic effectiveness and infection control.

The application of basic textbook principles to understanding economic behavior in the health-care industry is not a straightforward exercise because of the complex nature of health care as a service or product. Health care is not an item that is pulled off a store shelf, placed in a shopping cart, and paid for at the cash register. The desired result cannot be guaranteed and depends on various factors, many of which are beyond the control of the health-care provider. Economic analysis is based on the fundamental notion of efficient use of available resources. Two basic points are 1) economics is about resource allocation, and 2) efficiency in resource use (getting the most from available resources) in health care can be understood by identifying production functions representing health-care services.

Economics is a behavioral science that begins with two propositions about human behavior. First, human behavior is purposeful or goal directed, implying that persons act to promote their own interests. Second, human desires and demands are unlimited; however, resources are limited and cannot meet unlimited demands. Thus, the basic problem addressed by economics is how to allocate limited resources among unlimited demands. Within this context, the concept of cost in economics is based on opportunity costs rather than financial costs. Opportunity cost is the value of a resource when it is employed in its next best use. Costs are not expressed as expenses paid (or financial accounting) but as the value of lost output if resources were employed in an alternative productive process.

With the focus on resource allocation, one of the main concerns in designing a social mechanism to allocate society's resources is efficiency—getting the greatest output from productive inputs (a problem for suppliers). Another concern is product choice—determining what goods and services should be produced (meeting consumer demands). Finally, there is concern about product distribution (who gets the products produced).

Address for correspondence: Douglas Scott, Centers for Disease Control and Prevention, Mailstop A07, 1600 Clifton Road, Atlanta, GA 30333, USA; fax: 404-371-5078; e-mail: Dscott1@cdc.gov

### The Gold Standard of Resource Allocation Mechanisms

Understanding the social conditions that affect resource allocation is at the heart of economic thinking. Economics has what can be referred to as a "gold standard" of resource allocation mechanisms—the perfectly competitive market, which has the following characteristics (1): 1) many buyers and sellers with no single economic agent influencing the exchange of goods among market participants; 2) a homogeneous or standardized product (i.e., goods that individual producers cannot alter or differentiate to collect a higher price); 3) no barriers to movement of firms into or out of the market; 4) perfect information about market conditions that is available to all market participants; and 5) a fully defined system of property rights in which ownership of all products and productive resources is assigned.

This mechanism allows producers and consumers to freely interact; and from this interaction, consumer preferences about the product are revealed (Figure 1, demand curve), as well as the quantity producers are willing to supply at various prices (Figure 1, supply curve). The demand curve shows that consumers will purchase greater quantities of a good as price decreases, while the supply curve shows that

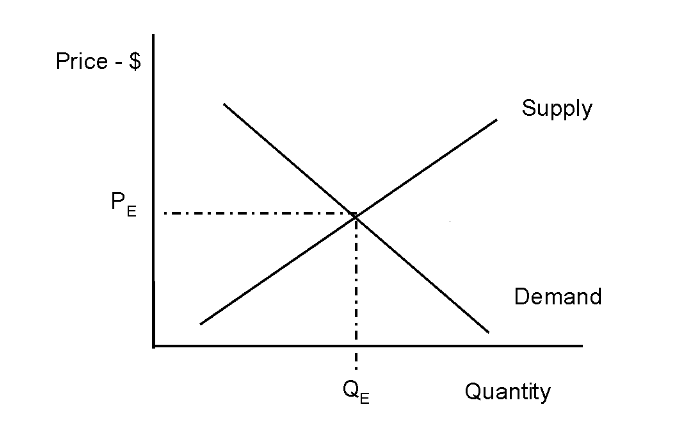

Figure 1. Supply and demand curves.

producers will produce greater quantities of a good as product price increases. As market participants interact, an equilibrium price level will emerge so that the quantity demanded at price $P_E$ by consumers is equal to the quantity that producers will supply at price $P_E$. $P_E$ becomes the market price because at no other price level does the quantity demanded by consumers match the quantity provided by suppliers. Prices greater than this level will result in excess supply; prices below this level result in excess demand.

Prices in a perfectly competitive market act as a feedback mechanism to market participants. Prices simultaneously reflect the value of the product to consumers and provide a signal to suppliers whether to change the amount of product they should produce relative to changes in consumer demand. The market for antibiotic drugs provides an example of how prices communicate preferences in the market place. There is debate regarding the extent to which prices for antibiotic drugs encourage the development and production of new agents to counter antibiotic resistance. An economist would assess this issue by examining the market price for antibiotics to determine whether prices are communicating to producers that new drugs are needed to meet future demands. If prices are not providing the appropriate "feedback," an economist would identify the characteristics in the market (e.g., number of producers, barriers to market entry or exit) responsible for the distortion in the price signal to market suppliers.

The power of the perfectly competitive market is that the perspectives of consumers, producers, and society as a whole converge. This market structure provides incentives for individual economic agents to act ultimately in the best interest of society (e.g., produce the greatest possible output from limited resources). Producers must be efficient and get the most output from the resources used. Inefficient producers will be unable to make a profit in the long run and will be forced to leave the market. Across the various markets, consumer demands are met (product choice), producers supply the most output possible (therefore maximizing profits), and society gets the most output from the scarce resources available.

Other types of resource allocation mechanisms are associated with markets with different characteristics, such as monopolies (single seller, e.g., power utilities) or oligopolies (a few sellers, e.g., automobile industry). However, these markets have shortcomings in terms of promoting the greatest output from society's resources and achieving the level of efficiency that could be obtained by the perfect market.

### Resource Allocation in Health Care

Examination of resource allocation in the health-care industry is complicated because the market characteristics differ from those in a perfectly competitive market. The market for health-care services is considered an imperfect market because—1) Health care is a heterogeneous product, as the patient can experience a range of outcomes; 2) Patients who are insured have third-party payers covering their direct medical expenses; and 3) A "market price" is lacking, i.e., no feedback mechanism exists that reflects the value of the resources used in health care.

While the perspectives of consumers, producers, and society converge in a perfectly competitive market, hospital patient costs in the health-care market are different for patients (consumers), health-care providers (suppliers), insurance companies (third-party payers), and society. The economic impacts of pain and suffering are of concern to the patient and society, but may not be relevant to a purely economic analysis of costs from the perspective of health-care providers or third-party payers (2).

Regardless of perspective, economic thinking provides one common goal: efficiency, or getting the most from available resources. A hospital administrator, for example, is faced with the challenge of organizing resources to meet the organization's goals. The relationship between the range of productive inputs utilized and outputs produced can be characterized by a production function, which shows the maximum amount of product that can be obtained from any specific combination of resources (or inputs) used in producing a product (or output). By identifying the relationship between output and inputs, one can find the combination of inputs and output that maximizes economic return.

The classic production function from economic theory follows a standard curve (Figure 2) that demonstrates the relation between one input and one output (3). This curve involves a variable input as opposed to a fixed input. Changes in the quantity of variable inputs will cause variation in the quantity of output produced (e.g., varying application of a fertilizer to a crop). Fixed inputs are those that must be in place before production can begin and do not vary with output levels (e.g., buildings). This curve embodies the notion of diminishing marginal returns. As one increases an input, a point is reached at which the additional output produced by adding another unit of input begins to get smaller and smaller, ultimately leading to a decline in the total output produced. The fixed input becomes overextended by the expanded production. For example, adding too much fertilizer to a crop can compromise soil quality and lead to a decline in output.

This is a technical relationship that does not yet include dollars. If the organizational goal is to maximize output, a producer would employ $I_B$ units of input to produce $O_B$ units of output. This approach would make sense if inputs were free. However, inputs are usually not free. This is where an economist steps in. At some point before the maximum, the value of the additional output created by an additional unit of input is less than the cost of this additional input (e.g., spending $10 in

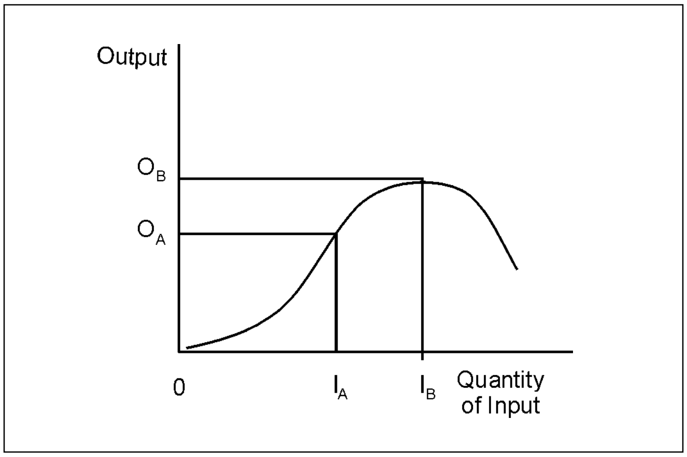

Figure 2. Standard curve of production function, demonstrating the relation between one input and one output.

additional input costs may yield only $8 in additional output value). The decision rule is to produce only as long as the value of additional output is just equal to the cost of the additional input.[1] For this figure, the region where it is "economic" to produce is somewhere between input quantities $I_A$ and $I_B$. The information needed to identify these productive relationships in a hospital must come from hospital epidemiologists as well as from hospital accountants. Epidemiology, being the principal measurement tool for population health status, provides measures of health-care outcomes (outputs). Measures of resource use (inputs) in a hospital should be based on hospital purchasing and cost accounting records (as opposed to hospital patient charges that do not accurately reflect actual resource use).

### Resource Use for Preserving Antibiotic Effectiveness

The framework for identifying efficient resource use can be applied to the production of health care in a hospital. Two major concerns of hospital epidemiologists are the effectiveness of antibiotic drugs and the incidence of health care-associated infections. Policy makers in health care are concerned about antibiotic resistance and how to maximize the effectiveness of existing antibiotic drugs. A production function quantifies the flow of resources that can be used to promote this effectiveness. Understanding the production function will help identify the trade-offs a clinician must make between the patient's health, the antibiotic treatment to prescribe, and the impact of this treatment on the rate of resistance. However, two production processes are affected by the decision to use antibiotics: promoting an individual patient's health and maintaining antibiotic effectiveness in the treatment of future patients.

The economic analysis in this instance is similar in complexity to the analysis of environmental problems such as air and water quality (4). Like clean air and clean water, antibiotic effectiveness is an economic good that is difficult to allocate efficiently using our gold standard allocation mechanism because it has some characteristics of a public good. Public goods represent a class of economic goods because by their nature they are nonrivaled and nonexclusive in consumption. The classic example of a public good often used by economists is national defense. It is unrivaled in consumption because, once provided, one person's consumption of defense does not affect another person's consumption. It is nonexclusive in consumption because, once provided, there is no practical way to exclude or prevent consumption of defense by those who choose not to pay for providing it. Because of these product characteristics, public goods will not work in our ideal resource allocation mechanism because there is no practical way to reveal a demand curve for a public good. Public goods are usually provided by a governmental agency (thus the name public good) or by some type of collective organization.

A continuum (Figure 3) can be used to describe the degree to which a particular economic good possesses characteristics that make it a private or public good. Antibiotic effectiveness falls between these two classes: it is exclusive in that only medical professionals (at least in the developed world) can administer the drug, but it is not purely nonrivaled because consumption of antibiotics by one person can affect future consumption by others.

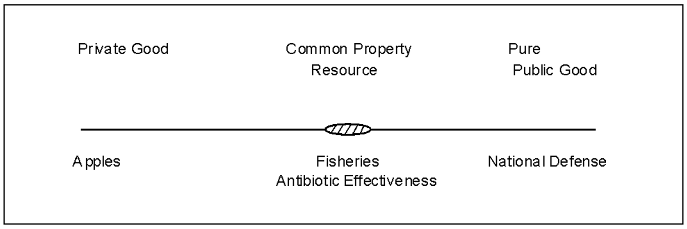

Figure 3. Continuum illustrating the degree to which an economic good has characteristics that make it a private or public good.

This leads to an externality: the use of a resource or product by one person can affect others without their permission. The decision to provide antibiotic treatment to one patient can affect the future efficacy and quality of the drug to other consumers (5). Resource allocation of antibiotic effectiveness is analogous to the management of fisheries: a fisherman, acting to maximize personal profits, can overfish and diminish the future stock (or quantity) of fish for all other fishermen of the same fish stock.

A fishery, like antibiotic effectiveness, is a common property resource. A common property resource, using fisheries as an example, is usually managed by some collective organization to restrict the quantity of fish harvested and monitor the health of the fishery to sustain a viable fish population in future years. Economists help design resource allocation mechanisms for common property resources that provide incentives (regulations, taxes, or subsidies) for individual agents to act in the interest of the whole collective. These incentives act like prices in that they provide the "feedback" about the values of the resources being used. To design a resource allocation mechanism for antibiotic effectiveness will necessitate much more information about the epidemiology and microbiology of biologic resistance and the trade-offs clinicians face in treatment decisions.

### Resource Allocation in Infection Control

The production function presented here is a simple relationship involving a single variable input. However, most production processes involve many variables, and determining the shape of a multidimensional production function can be a complicated statistical problem. However, understanding the technical relationship between health-care inputs (e.g., provider time, resources actually used for infection control) and outputs (i.e., patient health outcomes), and learning where resources are being over-employed (wth no real gains in output) are crucial in determining efficiency and therefore savings in production costs. Hospital infection control is an input to all the productive services a hospital provides (e.g., pediatric care, general surgery, trauma, cancer). Changes in infection control may influence health outcomes throughout the hospital, in ways that may not be obvious.

[1] A complicating factor omitted from the discussion is time. In a longer view of time, all fixed inputs are considered variable and can be redeployed to some other productive process. Therefore, fixed costs must be covered in the long run. Since fixed costs are "sunk" costs (spent before production even begins), it makes sense to keep operating for short time periods (as opposed to shutting down all production) if variable costs are covered.

### Conclusions

Efficiency in resource use (getting the most out of limited resources) is a goal that every health-care organization can accept, regardless of one's perspective (e.g., that of society, insurers, hospital administrators, or patients). Economic analysis is fundamentally about resource use and can serve an important role in health-care decision-making. Applying economic thinking to health care presents challenges to researchers and will require new approaches to analysis. Measuring the productive process in hospital care is complicated by the fact that the patient is both an input and an output in the process (i.e., the patient's health is a function of factors determined outside the hospital, such as lifestyle and genetics). Precise and accurate information from hospital epidemiology is critical to understanding the resources needed, and thus the economic impact, of caring for hospitalized patients.

Dr. Scott is a Steven M. Teutsch Post-Doctoral Fellow in prevention effectiveness methods, Division of Healthcare Quality Promotion, CDC. His areas of research include economic analysis of infectious disease prevention programs and the economic impacts of health care-acquired infections and antibiotic resistance.

### References

1. Mankiw NG. Principles of economics. Orlando: The Dryden Press; 1998.
2. Farnham PG, Ackerman SP, Haddix AC. Study design. In: Haddix AC, Teutsch SM, Shaffer PA, Duñet DO. Prevention effectiveness: a guide to decision analysis and economic evaluation. New York: Oxford University Press; 1996:12-26.
3. Mansfield E. Microeconomics: theory and applications. New York: W. W. Norton; 1982.
4. Kolstad CD. Environmental economics. New York: Oxford University Press; 2000.
5. Coast J, Smith RD, Millar MR. Superbugs: should antimicrobial resistance be included as a cost in economic evaluation? Health Economics 1996;5:217-226.
